# Nurses' Pension Funds

**Published:** 1887-02-26

**Authors:** Georgiana Bloomfield

**Affiliations:** 16, Holland Park


A STATEMENT has appeared in the *Daily Chronicle* of the 17th inst., which might lead to a misapprehension, therefore I must request you to correct it. It is stated in your article upon Nursing Institutions that Mr. Henry Burdett said, "They were within measurable distance of establishing a pension fund. Lady Bloomfield had promised a handsome sum as a nucleus." What I told Mr. Burdett, in an interview I had with him a fortnight ago, was, that "a Trained Nurses' Annuity Fund" was begun in 1874. Twelve annuities have been founded and above £4,000 invested. My wish has always been to enlarge this fund and make it the nucleus of a general fund, but I particularly told Mr. Burdett that I am only one of a committee, and can only act in concert with my colleagues, so it was impossible I could promise "a handsome sum" beforehand. Trusting you will publish this explanation,

I am, Sir,

16, Holland Park. GEORGIANA BLOOMFIELD.

